# Production of Value-Added Chemicals by *Bacillus methanolicus* Strains Cultivated on Mannitol and Extracts of Seaweed *Saccharina latissima* at 50°C

**DOI:** 10.3389/fmicb.2020.00680

**Published:** 2020-04-09

**Authors:** Sigrid Hakvåg, Ingemar Nærdal, Tonje M. B. Heggeset, Kåre A. Kristiansen, Inga M. Aasen, Trygve Brautaset

**Affiliations:** ^1^Department of Biotechnology and Food Sciences, Norwegian University of Science and Technology, Trondheim, Norway; ^2^Department of Biotechnology and Nanomedicine, SINTEF Industry, Trondheim, Norway

**Keywords:** macroalgae, *Saccharina latissima*, cadaverine, GABA, mannitol, laminaran, thermophilic, terpenoids

## Abstract

The facultative methylotroph *Bacillus methanolicus* MGA3 has previously been genetically engineered to overproduce the amino acids L-lysine and L-glutamate and their derivatives cadaverine and γ-aminobutyric acid (GABA) from methanol at 50°C. We here explored the potential of utilizing the sugar alcohol mannitol and seaweed extract (SWE) containing mannitol, as alternative feedstocks for production of chemicals by fermentation using *B. methanolicus*. Extracts of the brown algae *Saccharina latissima* harvested in the Trondheim Fjord in Norway were prepared and found to contain 12–13 g/l of mannitol, with conductivities corresponding to a salt content of ∼2% NaCl. Initially, 12 *B. methanolicus* wild type strains were tested for tolerance to various SWE concentrations, and some strains including MGA3 could grow on 50% SWE medium. Non-methylotrophic and methylotrophic growth of *B. methanolicus* rely on differences in regulation of metabolic pathways, and we compared production titers of GABA and cadaverine under such growth conditions. Shake flask experiments showed that recombinant MGA3 strains could produce similar and higher titers of cadaverine during growth on 50% SWE and mannitol, compared to on methanol. GABA production levels under these conditions were however low compared to growth on methanol. We present the first fed-batch mannitol fermentation of *B. methanolicus* and production of 6.3 g/l cadaverine. Finally, we constructed a recombinant MGA3 strain synthesizing the C30 terpenoids 4,4′-diaponeurosporene and 4,4′-diapolycopene, experimentally confirming that *B. methanolicus* has a functional methylerythritol phosphate (MEP) pathway. Together, our results contribute to extending the range of both the feedstocks for growth and products that can be synthesized by *B. methanolicus*.

## Introduction

Macroalgae, also denoted seaweeds, are explored as a potential sustainable biomass resource for microbial conversion into ethanol and butanol (Hou et al., 2015; Hou et al., 2017) and other platform chemicals, like succinic acid and 2,3-butanediol (Mazumdar et al., 2013; Bai et al., 2015; Marquez et al., 2015; Milledge and Harvey, 2016). The global production of seaweed reached 30.4 million tons in 2015, and cultivated seaweed constitutes more than 95% of the total production, with China as the biggest producer (FAO, 2018). Chile, China and Norway are the leading producers of wild species, mainly brown and red seaweed. Brown seaweeds are rich in structural (alginate and cellulose) and storage (laminaran and mannitol) carbohydrates (Schiener et al., 2015). The big brown algae are abundant in the North Atlantic Ocean, and technology for large-scale ocean farming is under development (Fossberg et al., 2018; Broch et al., 2019). So far, *Saccharina latissima* is the dominating cultivated species, and the free mannitol and laminaran comprise the easily extractable fraction of *S. latissima* (Horn et al., 2000a, b). These carbohydrates accumulate during the spring and summer, and thus fall is the best harvesting season for use as microbial cultivation feedstock (Schiener et al., 2015). Fermentation of seaweed-based mannitol extracts for bioethanol production by thermophilic *Clostridia* was recently described (Chades et al., 2018). Recombinant strains of the mesophilic bacteria *Escherichia coli* and *Corynebacterium glutamicum* are used for production of biofuels, chemicals and amino acids. *E. coli* can naturally assimilate both mannitol and glucose and has been engineered to produce 17.4 g/l of succinic acid from a hydrolyzate made from brown seaweed *Saccharina japonica* (Bai et al., 2015). Recently, the lysine producing strain *C. glutamicum* LYS-12 was engineered to assimilate mannitol and efficiently produce 2 g/l L-lysine from this substrate in shake flask experiments (Hoffmann et al., 2018). However, the high salt content of seaweed-based media represents a typical challenge in microbial cultivations and therefore strains with high salt tolerance are required.

**FIGURE 1:**
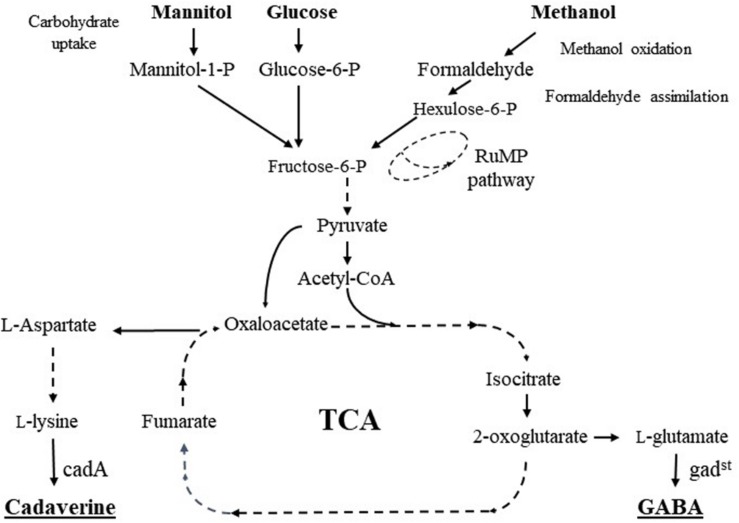
Schematic presentation of carbon metabolism and pathways for L-lysine, cadaverine, L-glutamate, and GABA biosynthesis in *B. methanolicus* (adapted from Müller et al., 2015). Broken lines indicate multiple reactions. Mannitol-1-P, mannitol-1-phosphate; Glucose 6-P, Glucose-6-phosphate; Fructose-6-P, fructose-6-phosphate, Hexulose-6-P, hexulose-6-phosphate; RuMP pathway, ribulose monophosphate pathway.

The biotechnological interest of the bacterium *Bacillus methanolicus* is largely linked to construction of genetically engineered cell factories that can convert the one-carbon (C1) compound methanol into useful products at elevated temperatures (Brautaset et al., 2010; Müller et al., 2015; Nærdal et al., 2015; Irla et al., 2016b). *B. methanolicus* MGA3 has proven to be a promising candidate for production of value-added chemicals from methanol, however *B. methanolicus* can also grow on mannitol as sole carbon and energy source. Still, no reports describe the use of mannitol as carbon source for production of industrially relevant chemicals by *B. methanolicus*. γ-Aminobutyric acid (GABA) and pentaene-1,5-diamine (cadaverine) are industrially important products originating from different precursors and biosynthetic routes; GABA is made from L-glutamate and branching out of the TCA cycle, while cadaverine is made from L-lysine in the aspartate pathway (Figure 1). Cadaverine is an important platform chemical especially due to its applicability in bio-based polyamide synthesis, by polymerization with dicarboxylic acids. Cadaverine is currently mainly fabricated by petroleum-based chemical synthesis and there is a growing interest in establishing alternative microbial routes for this chemical (Nærdal et al., 2015; Ma et al., 2017; Wang et al., 2018). GABA, naturally synthesized by plants and some microorganisms, has applications as food additive, as drug, and as constituent in biopolymers (Irla et al., 2016b; Xu et al., 2017).

The methylotrophic lifestyle of *B. methanolicus* has been extensively studied at the genetic, regulatory and physiological level (Schendel et al., 1990; Arfman et al., 1992; Heggeset et al., 2012; Müller et al., 2014; Irla et al., 2015). Growth rates of MGA3 on mannitol and methanol in shake flasks are similar and higher than on glucose (Jakobsen et al., 2006; Bozdag et al., 2015; Lopez et al., 2019), and mannitol growth has been used as an important reference to investigate and compare regulatory aspects (transcriptome and proteome, see below) associated with methylotrophic versus non-methylotrophic growth (Heggeset et al., 2012; Müller et al., 2014, 2015). Methylotrophic growth involves methanol oxidation by methanol dehydrogenase and formaldehyde fixation via the assimilatory ribulose monophosphate (RuMP) pathway (de Vries et al., 1992; Arfman et al., 1997; Brautaset et al., 2004; Heggeset et al., 2012; Krog et al., 2013). Energy and reducing power are mainly generated in the dissimilatory RuMP pathway, and not the TCA cycle. Non-methylotrophic growth on mannitol on the other hand does not involve the RuMP pathways, and energy and reducing power is dominantly generated in the TCA cycle. These differences have been demonstrated by transcriptome and proteome analyses showing that methanol oxidation, assimilatory and dissimilatory RuMP pathways, and TCA cycle genes and enzymes are differently regulated in cells growing methylotrophically versus non-methylotrophically (Heggeset et al., 2012; Müller et al., 2014, 2015). Moreover, several wild type strains of *B. methanolicus* were reported to grow well in the presence of 2% NaCl (Arfman et al., 1992), and strain PB1 has been adapted to rapid growth in artificial or natural seawater-based media (Komives et al., 2005). Thus, *B. methanolicus* displays several unique traits that makes it an interesting cell factory candidate for cultivations on seaweed-based growth media. We here explored the potential of *B. methanolicus* for utilizing seaweed extracts (SWE) containing mannitol and laminaran, prepared from *S. latissima* as an alternative and sustainable carbon source for production of useful chemicals. We report for the first time production of cadaverine, GABA and C30 terpenoids in MGA3 cells cultivated in a defined mannitol medium and in SWE medium.

## Results

### Preparation and Characterization of SWEs From *Saccharina latissima* Harvested in the Trondheim Fjord of Norway, for Cultivation of *Bacillus methanolicus*

To ensure a high carbohydrate content and to test for reproducibility, two batches of *S. latissima* seaweeds were harvested from the Trondheim Fjord in Norway in October 2014 and 2016. The SWEs were prepared by hot water extraction (1:1 dilution) at pH 3.7 and contained the soluble components of the raw material. The dilution was performed to enable separation of the liquid and solid phases due to the viscosity caused by the alginate in the biomass. The two SWE batches were similar with respect to dry weight and to content of laminaran, mannitol, and ash (Table 1). It has previously been shown that *B. methanolicus* MGA3 grows well in Man_10_ medium containing 10 g/l of mannitol as sole carbon source (Brautaset et al., 2004; Jakobsen et al., 2006) and thus the mannitol concentrations of 12–13 g/l in these SWEs should be sufficiently high to sustain growth. The measured conductivities corresponded to a salt content of ≤ 2% NaCl (32.3 mS/cm and 0.67 Osm/L), which is also below levels shown to be tolerable for growth of several strains of *B. methanolicus*, including strain MGA3 (Arfman et al., 1992; Pluschkell, 1998; Komives et al., 2005). The SWEs contain 60–70% more laminaran than mannitol. Although laminaran cannot be directly catabolized by *B. methanolicus* it represents a potentially interesting carbon source if hydrolyzed to glucose during extract preparations, and this was investigated further below. Summarized, the composition of the two SWEs were similar, with mannitol contents and conductivity suitable for *B. methanolicus* cultivation.

**TABLE 1:** Composition of SWE prepared from two batches of *Saccharina latissima* harvested in October in 2014 and 2016.

*S. latissima* batch (year)	Dry weight (g/L)	Laminaran^1^ (g/L)	Mannitol (g/L)	Ash (g/L)	Conductivity (mS/cm)	Osmolarity (Osm/L)
2014	60.2	21.5	12.5	19.8	27.9	0.68
2016	56.0	20.6	13.1	20.3	25.5	0.67

*^1^As glucose.*

### Establishment of Conditions for Cultivation of *B. methanolicus* Strains in SWE Based Growth Media

**FIGURE 2:**
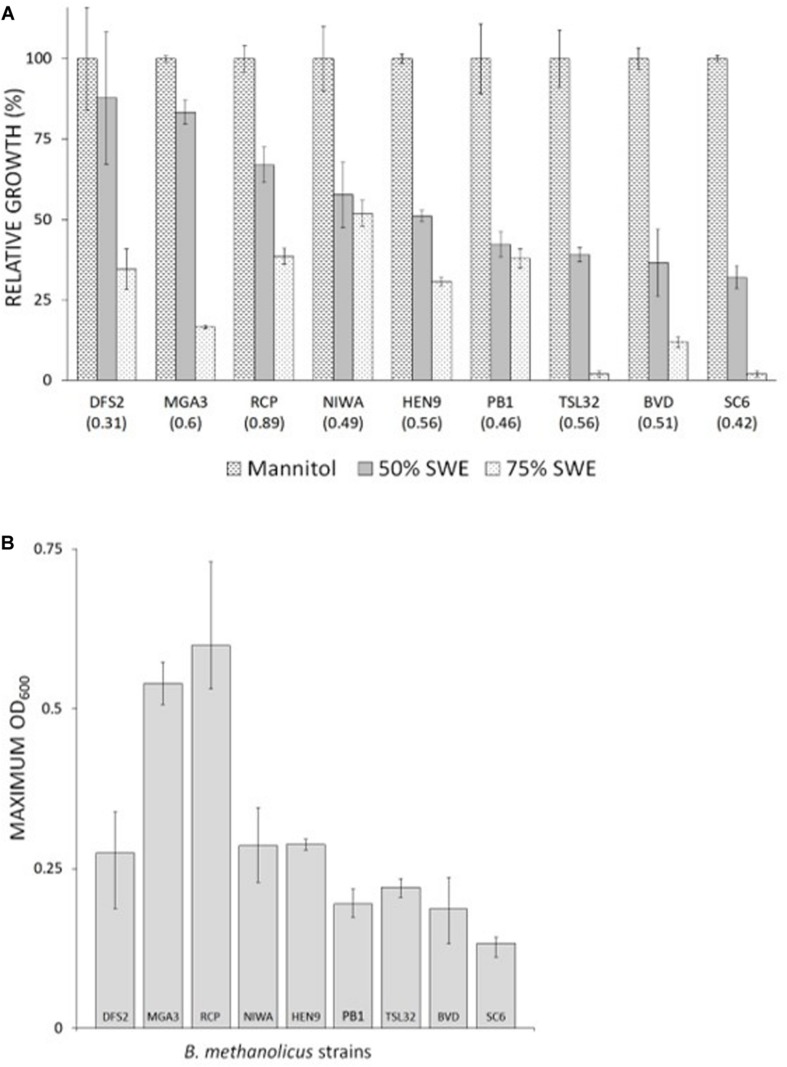
Tolerance of *B. methanolicus* wild type strains to grow in the presence of SWE. Cultivations were made in microwell plates for 20–24 h and with added mannitol to final concentration of 10 g/l. **(A)** Growth in the presence of 50 and 75% (v/v) SWE. 100% relative growth represent the maximum OD_600_ value obtained for each strain in Man_10_ medium. Obtained OD_600_ values in Man_10_ medium are given in parentheses for each strain. **(B)** Maximum OD_600_ values of *B. methanolicus* wild type strains in the presence of 50% (v/v) SWE. Maximum and minimum measured values for quadruplicate cultures are indicated.

Totally 11 different *B. methanolicus* wild type strains, DFS2, RCP, NIWA, HEN9, PB1, TSL32, BVD, SC6, N2, JCP, and CFS together with the well-characterized model strain MGA3 (see Table 2) were available. Based on previous characterizations indicating variations among these wild type strains both at the genetic (Brautaset et al., 2004; Heggeset et al., 2012) and the physiological level (Schendel et al., 1999), all of these strains were included in the initial testing under non-methylotrophic growth conditions, to fully explore the potential of this organism. Initial cultivations were performed in microwell plates with Man_10_-medium, and maximum (100%) OD_600_ values were recorded for each strain (values given in parentheses in Figure 2A). Strains CFS, N2, and JCP displayed poor or no growth under these conditions and they were not investigated any further. The remaining nine wild type strains displayed various growth properties, and they were next tested for growth with different amounts of SWE (0–90% v/v) added to the minimal medium. The aim of this experiment was to test tolerance of the *B. methanolicus* strains to the SWE with respect to its salt content as well as any other potentially growth inhibiting factors. To ensure similar and sufficient amounts of carbon source in all cultivations, mannitol was added to a final estimated concentration of 10 g/l for all media variants based on the mannitol content of the SWE (Table 1). All *B. methanolicus* strains reached different and lower OD_600_ values within 20–24 h incubation time in responses to increasing concentrations of added SWE, compared to when growing in Man_10_ medium (Figure 2A). Strains DFS2, MGA3, and RCP reached relatively high maximum OD_600_ values in the presence of 50% SWE, while PB1, TSL32, BVD, and SC6 displayed below 50% of their respective maximum OD_600_ values. All strains reached lower OD_600_ values in the presence of 75% SWE compared to 50% SWE (Figure 2A). Strain NIWA was least affected by high concentrations of SWE, reaching above 50% of the maximum OD_600_ value obtained in Man_10_ medium when cultivated in the presence of 75% SWE. Among the strains tested, RCP and MGA3 reached the highest maximum OD_600_ values within 20–24 h incubation with 50% SWE (Figure 2B). Summarized, *B. methanolicus* wild type strains displayed various growth tolerances to SWE, and strains RCP and MGA3 displayed the most promising growth properties in the presence of 50% SWE.

**TABLE 2:** Bacterial strains and plasmids used in this study.

Strain or plasmid	Description	Reference
*Escherichia coli* DH5α	General cloning host, F-*thi*-1 *endA*1 *hsdR*17 (r-,m-) *supE*44 _*lacU*169 (_80*lacZ*_M15) *recA*1 *gyrA*96 *relA*1	Stratagene
***Bacillus methanolicus***		
MGA3	Wild type strain	ATCC 53907
PB1	Wild type strain	ATCC 51375
BVD	Wild type strain	Brautaset et al., 2004
RCP	Wild type strain	Brautaset et al., 2004
HEN9	Wild type strain	Brautaset et al., 2004
SC6	Wild type strain	Brautaset et al., 2004
DFS2	Wild type strain	Brautaset et al., 2004
CFS	Wild type strain	Brautaset et al., 2004
JCP	Wild type strain	Brautaset et al., 2004
N2	Wild type strain	Brautaset et al., 2004
NIWA	Wild type strain	Brautaset et al., 2004
TSL32	Wild type strain	Brautaset et al., 2004
**Plasmid**		
pHP13	Cm^R^ and Em^R^, *E. coli/Bacillus* spp. shuttle vector	DSM 8773 (Haima et al., 1987)
pBV2mp-cadA	Km^R^ and ApR; pBV2mp derivative for *cadA* expression under control of the *mdh* promoter	Irla et al., 2016a
pTH1mp-gad^st^	Cm^R^; pTH1mp derivative for *gad* from *Sulfobacillus thermosulfidooxidans* DSM 9293 expression under control of the *mdh* promoter	Irla et al., 2016b
pTH1mp-lysC	pHP13 derivate with *lysC* under control of *mdh* promoter	Brautaset et al., 2010
pHYcrtMN	*B. subtilis* and *E. coli* shuttle vector; ori-pACYC177; ori-pAMα1; *crtM* and *crtN* genes of *S. aureus*; Amp^R^; Tc^R^	Yoshida et al., 2009
pTH1mp-crtMN	pHP13 derivative carrying *crtMN* from *S. aureus* under control of the *mdh* promoter	This study

### Cultivations of *B. methanolicus* Strains MGA3 and RCP in Sole SWE_50_-Medium

Based on the results from the microwell cultivations above, strains MGA3 and RCP were chosen for further testing of growth in minimal medium with 50% SWE (v/v) and no additional mannitol added, hereafter denoted as SWE_50_-medium. Growth in sole SWE_50_-medium was compared to growth in Man_10_ medium and these experiments were run in shake flasks to reach higher OD_600_ values and to enable measurements of growth rates. The results (Table 3) show that strains MGA3 and RCP grow in the SWE_50_-medium to maximum OD_600_ values of 6.0 ± 0.4 and 4.8 ± 0.4, respectively. The calculated growth rates of the two strains were 0.23 ± 0.03 and 0.41 ± 0.03 h^–1^, respectively. For MGA3 the growth rate in SWE_50_-medium was lower than in Man_10_-medium (0.38 ± 0.03 h^–1^), while the maximum OD_600_ value was higher in SWE_50_-medium relative to in Man_10_-medium. The cultivations of RCP in Man_10_ medium caused problems with cell aggregation impeding any reliable OD_600_ measurements (Table 3). The biological reason for this is unclear and was not investigated any further. SWE_50_-medium should contain ca 6 g/l of mannitol (see Table 1), and analysis of the growth media showed that approximately 5 g/L mannitol was consumed during these cultivations. Summarized, these results demonstrated that strain MGA3 displayed favorable properties for cultivations in SWE_50_-medium and Man_10_-medium and further experiments were therefore focused on this strain.

**TABLE 3:** Growth of strains MGA3 and RCP in SWE_50_-medium and Man_10_-medium.

	MGA3	RCP^1^
Maximum OD_600_ in Man_10_-medium	3.9 ± 0.1	ND^2^
Maximum OD_600_ in SWE_50_-medium	6.0 ± 0.4	4.8 ± 0.4
Growth rate in Man_10_-medium (h^–1^)	0.38 ± 0.03	ND^2^
Growth rate in SWE_50_-medium (h^–1^)	0.23 ± 0.03	0.41 ± 0.03

*^1^Media were supplemented with methionine. ^2^ND: not determined due to cell aggregation.*

### Cadaverine Production by Recombinant MGA3 (pBV2mp-cadA) in Man_10_ and SWE_50_ Medium Is Higher Than by Cells Growing in MeOH_200_ Medium

The previously constructed recombinant strain MGA3 (pBV2mp-cadA) expressing the lysine decarboxylase gene *cadA* from *E. coli* produces 10.2 g/l of the platform chemical cadaverine in fed-batch methanol fermentations (Nærdal et al., 2015). Cadaverine is a product derived from the aspartate pathway (see Figure 1) and it was therefore chosen as a model compound together with GABA (see below) for production analysis under methylotrophic versus non-methylotrophic growth. MGA3 (pBV2mp-cadA) cells were cultivated in shake flasks with Man_10_, SWE_50_, and MeOH_200_-medium and cell cultures were analyzed for cadaverine production (Table 4). Interestingly, cadaverine production titers in both Man_10_ (338 ± 6 mg/L) and SWE_50_ (287 ± 13 mg/L) media were higher than the production titers obtained from cells growing in MeOH_200_-medium (240 ± 1 mg/L). Like for the MGA3 host strain, the growth rate of MGA3 (pBV2mp-cadA) in SWE_50_-medium was low compared to in Man_10_-medium (data not shown).

**TABLE 4:** Production levels (mg/L) of GABA and cadaverine by recombinant *B. methanolicus* MGA3 strains cultivated in Man_10_, SWE_50_, and MeOH_200_ media.

Strain name	Growth medium			
	Product	MeOH_200_	Man_10_	SWE_50_
MGA3 (pTH1mp-gad^*st*^)	GABA	260^1^	152 ± 4	13 ± 1
MGA3 (pBV2mp-cadA)	Cadaverine	240 ± 1	338 ± 6	287 ± 13

*Cells were cultivated in shake flasks and production was measured after 24–29 h. ^1^Reported production after 20–40 h cultivation. Data adapted from Irla et al. (2016b).*

### Production of GABA by Recombinant MGA3 (pTH1mp-gad^*st*^) in Man_10_ and SWE_50_ Medium Is Low Compared to in MeOH_200_ Medium

*Bacillus methanolicus* MGA3 is a native L-glutamate producer (Brautaset et al., 2003) and the recombinant strain MGA3 (pTH1mp-gad^*st*^) expressing the glutamate decarboxylase gene *gad*^*st*^ from *Sulfobacillus thermosulfidooxidans* produce up to 9 g/l of GABA in fed-batch methanol fermentations (Irla et al., 2016b). Omics analyses have shown that regulation of genes and enzymes representing in particular the RuMP and TCA cycles (see Figure 1) is largely different in MGA3 cells growing methylotrophically vs. non-methylotrophically (Jakobsen et al., 2006; Heggeset et al., 2012; Müller et al., 2014, 2015). Therefore, it was of interest to analyze production levels of GABA, which branches out of the TCA cycle, upon cell growth in Man_10_ and SWE_50_, in comparison to MeOH_200_ medium. By including both Man_10_ and SWE_50_ media any effects of the carbon source could be distinguished from other effects related to the complex SWE_50_ medium. MGA3 (pTH1mp-gad^*st*^) cells were first cultivated in shake flask in Man_10_ medium and the results (Table 4) showed that this strain produced 152 mg/L GABA, which is about 60% of the production level obtained using methanol medium (260 mg/L after 20–40 h) during shake-flask cultivation (Irla et al., 2016b). Surprisingly, when cultivated in SWE_50_-medium the cell growth was poor and GABA production was only 13 mg/L corresponding to 9% of the production obtained in Man_10_ medium. Together, these results demonstrated that *B. methanolicus* MGA3 (pTH1mp-gad^*st*^) can produce GABA upon non-methylotrophic growth in Man_10_ and SWE_50_ medium, however the production levels are low compared to when the strain is grown methylotrophically in MeOH_200_ medium.

### MGA3 (pBV2mp-cadA) Secretes 6.3 g/l of Cadaverine Under Fed-Batch Mannitol Fermentation

To date, no fermentation data for non-methylotrophic conditions have been published for *B. methanolicus*, and therefore cadaverine production of strain MGA3 (pBV2mp-cadA) was tested in high cell density fed-batch mannitol fermentation. A robust protocol for mannitol feeding was established, and the mannitol concentration was monitored by HPLC. The recombinant cell culture reached a cell dry weight of about 55% compared to the biomass typically obtained under analogous methanol fermentations (Table 5). The highest cadaverine production titer achieved was 6.3 g/l (concentration corrected for dilution due to the feeding) corresponding to ∼60% of the previously reported production level obtained during fed-batch methanol fermentation (Irla et al., 2016a). Interestingly, the calculated production yield per cell dry weight was higher on mannitol (0.19 g/g CDW) than on methanol (0.17 g/g CDW).

**TABLE 5:** Cadaverine production in fed-batch methanol and mannitol fermentations of *B. methanolicus* MGA3 (pBV2mp-cadA).

	Carbon source	
	Mannitol^3^	Methanol^1^
Cadaverine (g/L)	6.3 ± 0.9	10.2 ± 1.2
Cell dry weight (CDW) (g/L)^2^	34.2 ± 1.2	60.9 ± 1.3
Cadaverine yield (g/g CDW)	0.19 ± 0.02	0.17 ± 0.02

*^1^Values imported and adapted from Irla et al. (2016a). ^2^CDW, cell dry weight, maximum values from stationary growth phase are given. ^3^All values are corrected for the dilution due to feeding, with a factor of 3.3.*

### Hydrolysis of the Laminaran Fraction to Glucose Increased the Total Available Carbohydrate Content of the SWE for Cultivations of *B. methanolicus*

**FIGURE 3:**
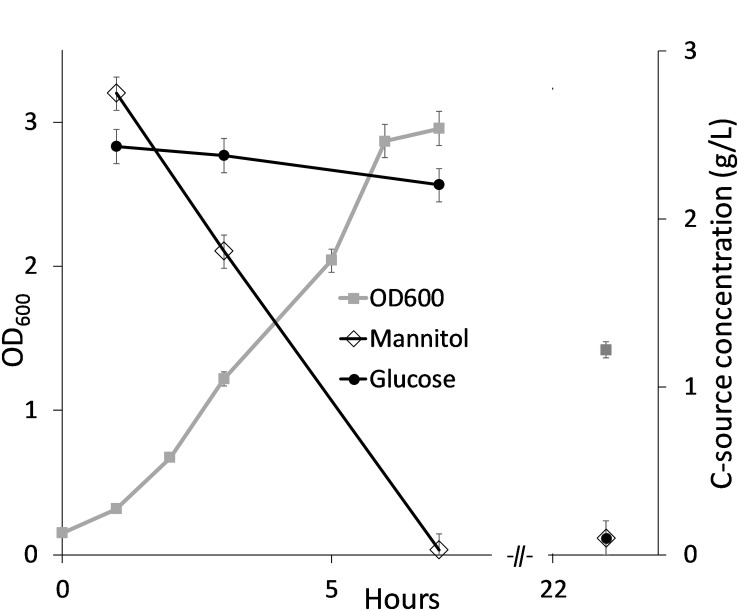
*Bacillus methanolicus* MGA3 cultivated in MVcMY minimal medium with mannitol and glucose (2.5 g/L each). Consumption of mannitol and glucose was measured over time and shown as remaining sugar in culture medium. Maximum deviation for triplicate cultivations is presented.

In order to establish fed-batch fermentations of *B. methanolicus* in SWE based medium, a higher concentration of carbon source is required to ensure high cell densities. It was therefore investigated if and how the laminaran fraction would be utilized by *B. methanolicus* cells if hydrolyzed to glucose monomers prior to cultivation. *B. methanolicus* MGA3 co-utilizes mannitol and methanol when both carbon sources are present (Jakobsen et al., 2006). MGA3 was therefore tested for growth in MVcMY minimal medium with both mannitol and glucose added (2.5 g/L each), and the results showed that mannitol and glucose were consumed sequentially, with mannitol as the preferred substrate (Figure 3).

**FIGURE 4:**
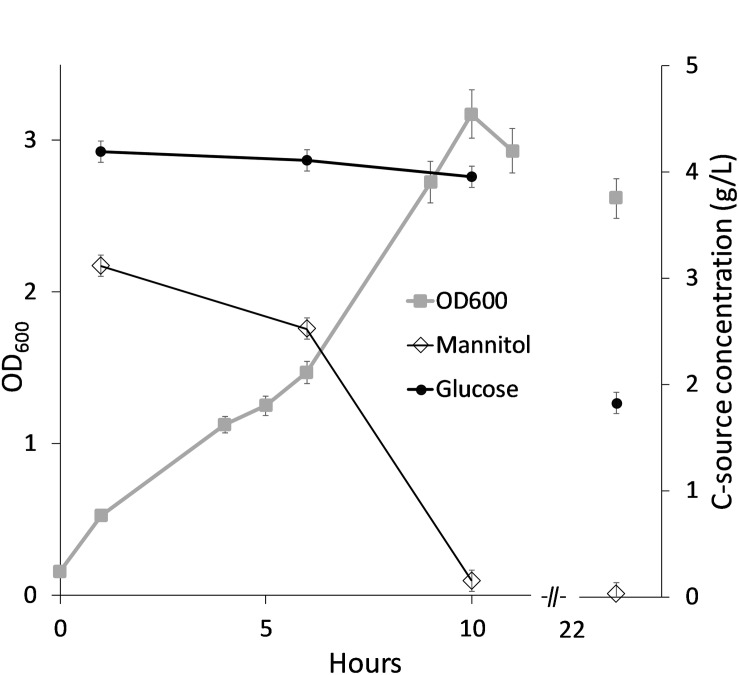
*Bacillus methanolicus* MGA3 cultivated in SWE2_20_ medium with hydrolyzed laminaran. Consumption of mannitol and glucose was measured over time and shown as remaining sugar in culture medium. Maximum deviation for triplicate cultivations is presented.

Motivated by this, an alternative SWE medium was prepared where the laminaran was hydrolyzed to glucose (denoted SWE2) and tested as growth medium for *B. methanolicus* MGA3 in shake flasks. These cultivations were performed with 20% SWE2 (SWE2_20_ medium) corresponding to mannitol and glucose concentrations of approximately 3 and 4 g/L, respectively. 20% SWE2 was chosen, to provide approximately 2.5 g/L of mannitol, and facilitate utilization of both carbon sources in the medium. The results showed that MGA3 grows well on this medium and that the mannitol and glucose components were utilized sequentially as expected (Figure 4).

Analysis of the growth medium at the end of the cultivation showed that ca 5 g/L of the total available carbon (mannitol and glucose) was consumed. Mannitol was exhausted in cultures after 7 and 10 h in Figures 3, 4 respectively. It is not known whether the glucose consumed after these timepoints resulted in increased cell density (no datapoints). However, as shown for cultivation in SWE2_20_ medium (Figure 4) there was still glucose left in the culture medium after 24 h, indicating that the glucose is primarily utilized for maintenance of viable cells.

### Heterologous Expression of Dehydrosqualene Synthase and Dehydrosqualene Desaturase Results in Production of C30 Terpenoids by *B. methanolicus*

**FIGURE 5:**
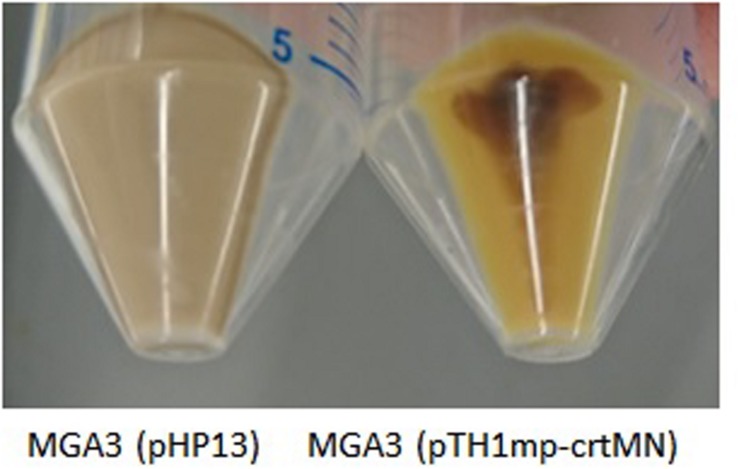
Cell pellets of recombinant *B. methanolicus* MGA3 (pHP13) and MGA3 (pTH1mp-crtMN) strains cultivated in SWE_50_ medium.

In addition to exploring the substrate range of *B. methanolicus* it is of interest to also extend the range of value-added products that can be produced at 50°C. Microbial production of terpenoids, and in particular C40 carotenoids, has gained increased interest as earlier reviewed (Lee and Schmidt-Dannert, 2002; Das et al., 2007; Niu et al., 2017). In most prokaryotes, terpenoids are produced via the methylerythritol phosphate (MEP) pathway (Rohmer et al., 1993). Inspection of the *B. methanolicus* MGA3 genome sequence indicated that it has all genes necessary for a complete MEP pathway for the biosynthesis of the terpenoid precursors isopentenyl pyrophosphate (IPP) and dimethylallyl pyrophosphate (DMAPP), however the biological function of this putative biosynthetic pathway has until now not been experimentally confirmed. To verify that the identified genes are active, *Staphylococcus aureus* genes *crtM* and *crtN* encoding dehydrosqualene synthase and dehydrosqualene desaturase, respectively, were cloned into vector pTH1mp under the control of the *mdh* promoter, yielding plasmid pTH1mp-crtMN. Yoshida et al. (2009), reported that heterologous expression of *crtM* and *crtN* in *B. subtilis* results in the production of two different C30 terpenoids, diaponeurosporene, and diapolycopene providing yellow pigmentation of the recombinant cells. The plasmid pTH1mp-crtMN was transferred into *B. methanolicus* MGA3 and the resulting strain MGA3 (pTH1mp-crtMN) together with the control strain MGA3 (pHP13) were tested for production of the terpenoids during cultivations on Man_10_, SWE_50_, and MeOH_200_ media. Yellow pigmentation of the MGA3 (pTH1mp-crtMN) cell pellets was obtained under all three growth conditions, indicating production of the expected terpenoids (Figure 5).

The presence of terpenoids was analyzed by adsorption spectrometry of acetone extracts prepared of the lysed pellets of MGA3 (pTH1mp-crtMN). Three absorbance maxima were detected in the extracts, in agreement with terpenoid presence (data not shown). No terpenoids were detected in extracts of the control strain MGA3 (pHP13).

**FIGURE 6:**
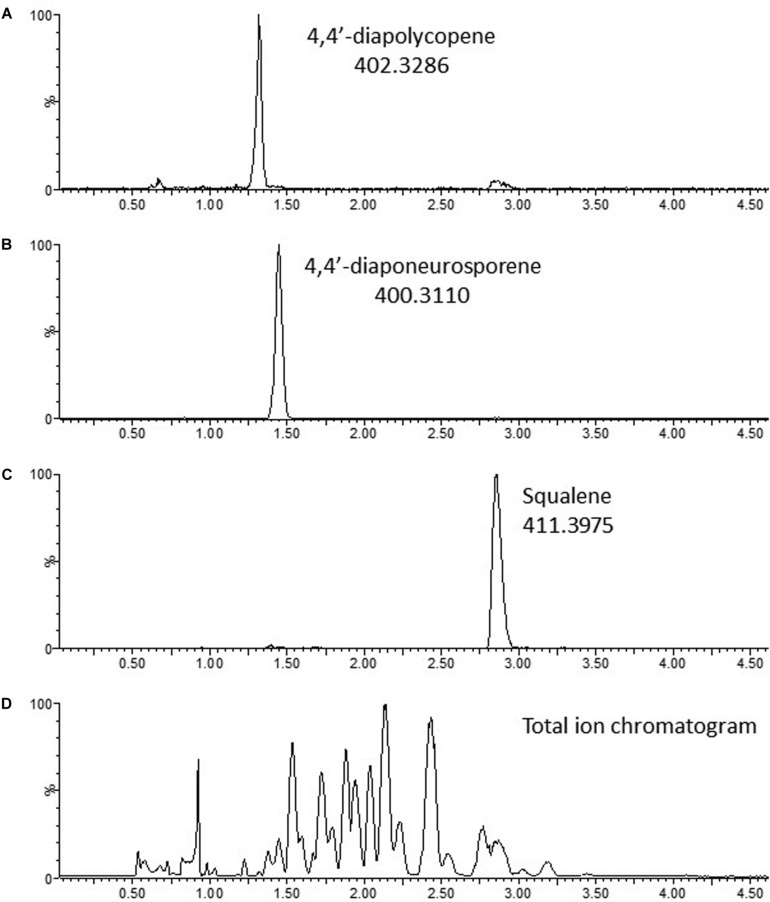
Chromatograms of terpenoids produced by *B. methanolicus* MGA3 (pTH1mp-crtMN). **(A)** 4,4′-Diapolycopene; **(B)** 4,4′-Diaponeurosporene; **(C)** Squalene; **(D)** Total ion chromatogram. Continuum data.

To characterize the compounds produced, acetone extracts of MGA3 (pTH1mp-crtMN) cell pellets were analyzed by liquid chromatography quadrupole time of flight (LC-qTOF). Monoisotopic masses observed, m/z 402.3284 and m/z 400.3127, correspond to the calculated monoisotopic masses of 4,4′-diapolycopene and 4,4′-diaponeurosporene (402.32870 and 400.31300 Da) respectively, with a mass error of 0.3 mDa (centroid data). Chromatograms are shown in Figure 6, and mass spectra are shown in Supplementary Figure 1. In addition, the theoretically calculated isotope patterns coincide with the isotope patterns obtained for the two compounds with a confidence fit of 99.99 and 99.97% for 4,4′-Diapolycopene and 4,4′-Diaponeurosporene, respectively.

Together, these results showed that MGA3 has a functional MEP pathway that can be engineered to produce C30 terpenoids, indicating that *B. methanolicus* has a potential as platform strain for production of terpenoids from sustainable raw materials at elevated temperatures.

## Discussion

In this work SWEs from the brown algae *S. latissima* harvested at the Norwegian coast were prepared and shown to contain substantial concentrations of the carbohydrates mannitol and laminaran. We demonstrated that the SWEs can be used as an alternative and non-methylotrophic raw material for cultivations of *B. methanolicus* strains at 50°C. Out of 12 different wild type strains tested, strain MGA3 possessed the most favorable growth properties, tolerating up to 50% SWE (v/v) in the growth medium. The biological reason for why the SWE concentration could not be further increased (to 75%) without negatively affecting growth is not known. The increased salt content could possibly contribute to this effect, however other extracted compounds such as phenols could also impose a negative effect on cell growth (Daglia, 2012; Pérez et al., 2016). The polyphenol content of the seaweed will likely vary between the seasons, where a higher content in fall than spring has been reported for *S. latissima* (Roleda et al., 2019).

To date, no fermentation production data on carbon sources other than methanol has been reported for *B. methanolicus*, and we present the first fed-batch mannitol fermentation of recombinant *B. methanolicus* MGA3 (pBV2mp-cadA) secreting 6.3 g/l of cadaverine which is about 60% of the titer previously reported for fed-batch methanol fermentation of this strain (10.2 g/l). Interestingly, the production yield (0.19 g/g CDW) on mannitol is higher than on methanol (0.17 g/g CDW). Mannitol is easily facilitated by *B. methanolicus* and it is not associated with any toxic degradation intermediates such as formaldehyde made by oxidation of methanol under methylotrophic growth (Jakobsen et al., 2006).

Shake flask cultivations of *B. methanolicus* MGA3 in SWE_50_ medium, and production of the C30 terpenoids 4,4′-diaponeurosporene and 4,4′-diapolycopene, and platform chemicals GABA and cadaverine by genetically engineered MGA3 strains under such conditions was here demonstrated. Notably, the production titer by strain MGA3 (pBV2mp-cadA) in SWE_50_ medium (287 mg/l) was slightly higher than on methanol medium (240 mg/l). Several bacterial species have been genetically engineered for overproduction of GABA and cadaverine. Production titers up to 125 g/L of cadaverine in sugar-based fed-batch fermentations have been reported for recombinant *C. glutamicum* strains (Kim et al., 2020). The highest reported production titer of GABA was achieved with recombinant *E. coli* strains producing up to 308.96 g/l of this compound on sugar-based media and with the GABA precursor L-glutamate added in excess (Ke et al., 2016; Xu et al., 2017). *Lactobacillus brevis* strains have been constructed and reported to display GABA production titers up to 201 g/L for resting cells (Shi et al., 2017). Based on all this, both GABA and cadaverine production titers by recombinant *B. methanolicus* need to be increased to be industrially competitive. The small-scale results presented here demonstrate a potential of using *B. methanolicus* strains for more sustainable production of platform chemicals and terpenoids from SWEs at elevated temperatures, and thus expands the application range of this organism as a versatile cell factory for industrial biotechnology.

Hydrolysis of the laminaran fraction of the SWE (i.e., SWE2) and the concomitant utilization by *B. methanolicus* MGA3 of both the mannitol and the glucose components in a sequential manner was demonstrated. It is unknown whether the consumed glucose was used for cell growth, but the remaining glucose in the culture medium by the end of incubation, indicated that this carbon source might primarily be used for maintenance of viable cells under these growth conditions. The same total amount of the carbon sources (5 g/L, corresponding to approximately 160 mmol carbon) was consumed in both cultivations in Man_10_ (mannitol) and SWE2_20_ (mannitol + glucose) medium. To fully exploit the carbon sources available in the SWEs, glucose utilization could possibly be increased by overexpression of genes involved in glucose uptake and metabolism. A further optimization of the glucose utilization was however not within of the scope of this work.

In addition to the economical aspect, such as cost of the biomass, the high water content and resulting low concentration of easily fermentable sugars represent a technical limitation for future use of seaweed biomass as a feedstock for fermentation industry. In the batches of *S. latissima* used in our work, the content of laminaran and mannitol constituted ∼36% of dry weight, or 68 g/kg fresh biomass, which are representative concentrations for this species when harvested in the autumn. For industrial fermentation processes, the carbon source concentration should preferably be higher, and this could in principle be achieved by fermentation of the whole slurry, concentrating the extract by evaporation or membrane filtration, or by using dried biomass. However, as shown in this work, *B. methanolicus* growth was reduced when the SWE concentrations (1:1 diluted) were increased from 50 to 75%, implying that further processing of the SWEs and/or strain adaptation is needed to enable growth on more concentrated SWEs in industrial fermentations using this organism. Tolerance to higher salt concentrations has previously been obtained by evolutionary adaptation for *B. methanolicus* strain PB1, to allow growth in seawater-based media (Komives et al., 2005). As an alternative to increasing the amount of SWE to increase the concentration of carbon source, the extract could potentially be used as a source of minerals and micronutrients in fermentation (Sharma et al., 2018). In a study by Allahgholi et al. (2020), *B. methanolicus* MGA3 was cultivated on extracts of the alternative seaweed *Laminaria digitata*. In that study, it was found that reducing the concentration of the SWE and supplementing the culture with extra mannitol, resulted in higher biomass formation than when the strain was cultivated on a reference medium without SWE, and with mannitol as sole carbon source. As shown here, *B. methanolicus* utilizes both mannitol and glucose when cultivated with a mixture of the two carbon sources, thereby allowing a possible application of the SWE’s as a growth promoting supplement in industrial fermentations. These results contribute to future sustainable bioprocesses based on seaweed-supplemented growth media. However, further work must be done in development of more effective methods for large-scale preparation of SWE media to make this experimentally possible.

## Materials and Methods

### Preparation of Seaweed Extract

Frozen, milled *S. latissima* (two batches, harvested October 2014 and 2016, respectively, from the Trondheim Fjord, Norway) was thawed at 4°C over night. The extracts (SWEs) were prepared according to the protocol developed by Horn et al. (2000a), but using pH ∼3.5, since later works have shown that the laminaran becomes less available for enzymatic hydrolysis after extraction at pH < 3 (Adams et al., 2008; Sandbakken et al., 2018). Briefly, hot water (60–70°C) was added to the biomass (biomass:water, 1:1, weight basis), and pH was adjusted to ∼3.5 by addition of 1M H_2_SO_4_, before incubation at 70°C for 1 h to completely dissolve laminaran. The slurry was centrifuged when still hot (>60°C) (7000 rpm, 12220 × G, 15 min). The supernatant was sieved, and the pH was adjusted to pH 5.0 for enzymatic hydrolysis of laminaran or to pH 6.5–7 for cultivations without any laminaran hydrolysis.

For laminaran hydrolysis, 575–885 μl of the enzyme Cellic CTect2 (SAE0020 Sigma) was added per 100 ml SWE, followed by incubation with rotation at 50°C for 16–18 h. After incubation, the pH of the enzyme-treated extracts was adjusted to 6.5–7. The resulting extracts (SWE2) were frozen at −20°C, and either autoclaved or sterile filtered (0.22 μm) before use in shake flask and microwell cultivations.

Extract without added enzyme was treated in parallel and used as control in growth experiments. The conductivities and osmolarities of the extracts were determined using a Sension + MM 374 Laboratory meter (Hach, Loveland, CO, United States) and an Osmomat 030-D (Gonotec GmbH, Berlin, Germany), respectively.

### Biological Materials and Growth Conditions

The bacterial strains and plasmids used in this study are listed in Table 2. *E. coli* DH5α was used as cloning host and cultivated at 37°C in LB medium or on LA plates (Bertani, 1951) supplemented with chloramphenicol (25 μg/ml) or kanamycin (25 μg/ml). *B. methanolicus* strains were cultivated at 50°C in MVcMY minimal medium with 200 mM methanol (MeOH_200_) as previously described (Brautaset et al., 2004; Jakobsen et al., 2006). MeOH_200_ medium contains 0.025% yeast extract, supporting a biomass production equivalent to OD_600_ ≈ 0.04 in microwell cultivations. Mannitol and seaweed cultivations were performed with the same medium, replacing the methanol with 10 g/L mannitol (Man_10_-medium), SWE (0, 25, 50, 75, or 90% v/v), or SWE with hydrolyzed laminaran (SWE2) (10, 20, 30, or 50% v/v). The SWE media were named after the amount of SWE added, e.g., SWE_20_ contains 20% SWE and SWE_50_ contains 50% SWE. The pH was re-adjusted to pH 7.2 with 1M NaOH after mixing of minimal medium and SWE. Insoluble material was pelleted by centrifugation, and the supernatant was used as growth medium. Conductivity and carbohydrate content of the medium were measured before and after removal of insoluble material. The SWE was either autoclaved or sterile filtered before use in cultivation experiments. For initial growth experiments with SWE, the medium was supplemented with mannitol to yield a final concentration of 10 g mannitol/L.

Amino acids, L-methionine (1.5 mM), L-leucine (1 mM), L-threonine (1 mM) were added for growth of specific wild type strains, based on previously characterized L-methionine (DFS2, RCP, HEN9, TSL32, JCP, and N2), L-threonine (TSL32 and JCP), and/or L-leucine (N2) auxotrophies of the different *B. methanolicus* wild type strains (Schendel et al., 1999; Brautaset et al., 2004). For GABA production, recombinant strains were cultivated in MVcMY minimal medium with 20 μM pyridoxal 5′-phosphate (PLP), and reduced magnesium concentration (0.04 mM) as previously described (Irla et al., 2016b). A carbon source was added as appropriate. All media were supplemented with chloramphenicol (5 μg/ml) or kanamycin (25 μg/ml) when appropriate.

Cultivations of *B. methanolicus* were performed as previously described (Brautaset et al., 2004; Jakobsen et al., 2006), with minor changes. Precultures were centrifuged and resuspended in fresh medium before inoculation of production cultures. Growth was performed either in 96-well plates (100 μl) at 900 rpm and with 75% relative humidity, or in 250 ml baffled shake flasks (40 ml) at 200 rpm. Growth was monitored by measuring the optical density at 600 nm (OD_600_), with either a Unicam Helios Epsilon (shake flask cultivations) or a Spectramax Plus 384 (microwell plates). All cultivations were performed in triplicates (shake flasks) or minimum quadruplicates (microwell plates).

### Construction of Plasmid pTH1mp-crtMN

Plasmid DNA was isolated by the Wizard Plus SV Minipreps (Promega). For construction of vector pTH1mp-crtMN, plasmid pTHmp-lysC (Brautaset et al., 2010) was digested with *Acc*65I and *Pci*I and used as backbone. A DNA fragment corresponding to the crtMN was PCR amplified from pHYcrtMN (Yoshida et al., 2009) using the following primers:

Forward primer (crtMN-R):

5′-TTTTGGTACCTTATACGCCCCGCTCAATAT CTTT-3′
(Recognition site for *Acc*65I is underlined).

Reverse primer (crtMN-F):

5′-TTTTACATGTGACAATGATGGATATGAATTTTAA ATATTG-3′
(Recognition site for *Pci*I is underlined).

The resulting PCR-product (2.4 kb) was end digested with *Acc*65I and *Pci*I, and ligated into the backbone, resulting in the vector pTH1mp-crtMN (8.2 kb). The plasmid was verified by DNA sequencing.

Preparation of electrocompetent cells of *B. methanolicus* and transformation thereof was performed as previously described (Jakobsen et al., 2006). SOBsuc plates (1% (w/v) agar) supplemented with suitable antibiotics were used instead of regeneration plates, as described by Irla et al. (2016a).

### Extraction and Analysis of Terpenoids From *B. methanolicus*

50 ml culture samples were collected and centrifuged at 4000 rpm for 10 min at 25°C. Pellets were washed with 2 ml TE buffer (10 mM Tris-HCl, 1 mM EDTA, pH = 8.0) and resuspended in 200 μl TE buffer. Extraction was performed as previously described by Yoshida et al. (2009) with minor modifications. 20 ul squalene (≥98%) was added as internal standard, and cells were lysed with 50 μl lysozyme (20 mg/ml) at 37°C for 15 min. Terpenoids were extracted with 500 μl acetone. Samples were mixed (2 min) and heated to 55°C for 15 min with mixing every 5 min. The samples were centrifuged at 4500 rpm for 3 min and the supernatant collected in a clean tube. Absorbance was measured in cuvette on Infinite 200 Pro plate reader (Tecan Group, Ltd.) in the spectrum 350–530 nm with a data interval of 1.0 nm. For UHPLC analyses, extracts were evaporated under N_2_-gass and resuspended in 1/10 volume acetone (10X concentrated).

### Analysis of Terpenoids by Ultra-High-Performance Chromatography (UHPLC) Coupled With Quadrupole Time of Flight (qTOF) Mass Spectrometry

Analyses were performed with an ACQUITY UPLC system coupled to a Synapt G2Si HDMS mass spectrometer (Waters, Milford, MA, United States) equipped with an APCI source operating in positive mode. UHPLC–qTOF data were acquired and processed using MassLynx software (v4.1).

The chromatographic column applied was a Waters ACQUITY UPLC CSH C18 (130 Å, 150 mm × 2.1 mm L × I.D. 1.7 μm). Column manager was set to 40°C. Mobile phases consisted of (A): acetonitrile (80%)/methanol (15%)/isopropanol (5%) with 0.1% formic acid and (B): isopropanol (90%)/acetonitrile (10%) with 0.1% formic acid. Separation was performed isocratic at 100% mobile phase A with a flow rate of 0.65 mL/min until 4 min when a washout step was initiated. The flow rate was reduced to 0.15 mL/min with 50% A within 0.1 min and kept for 2 min, before the system was returned to initial conditions (equilibration) within 1 min and kept for one additional minute. Total run time was 8 min. The injection volume was in the range 2–10 μL, with acetone as solvent for all samples.

MS analyses were performed under constant APCI conditions. Parameters were adjusted as follows: the corona voltage and source offset voltages (2.5 kV and 80 V), cone voltage (30 V), source temperature (120°C), desolvation gas temperature (500°C), and desolvation gas flow (1000 L/h). The cone gas flow rate (150 L/h), and the nebulizer gas flow (6 bar). Samples were analyzed in scan mode. Mass range was set to 50–1200 Da, as in the valid calibration performed with Na-formate just prior to analysis. Data was collected in high-resolution mode with 0.2 s scan time. During the analysis, a lock mass of 10 μL/min leucine enkephalin (800 pg/mL) was infused into the ion source through a separate capillary (2.5 kV) to correct the mass axis on the fly. Compounds 4,4′-diapolycopene and 4,4′-diaponeurosporene were observed as M^+^ ions. Squalene was observed as an M + H^+^ ion. Isotope patterns were calculated by Elemental Composition in MassLynx v4.1

### Fed-Batch Mannitol Fermentation of *B. methanolicus*

Fed-batch fermentation was performed in a slightly modified UMN1 medium using Applikon 3 L fermentors with an initial volume of 0.75 L medium (Jakobsen et al., 2009). The initial batch medium contained 20 g/l mannitol as carbon source and kanamycin (30 μg/mL) or chloramphenicol (5 μg/mL) was added as appropriate. Shake flask cultures in mannitol medium were used as inocula and were harvested at an OD_600_ of 1.2 to 2.5. The fermentors were inoculated to OD_600_ of 0.1. Fermentations were performed at 50°C and the pH was maintained at 6.5 by automatic addition of 12.5% (w/v) NH_3_ solution. The initial agitation speed was set to 600 rpm and the initial aeration rate as 0.5 l/min. The dissolved oxygen level was maintained at 30% saturation by increasing the agitation speed up to 2000 rpm and increasing the air flow up to 1 l/min.

The mannitol concentration in the fermentation broth was maintained at 5–25 g/l by adjusting the feeding rate. The mannitol feed solution contained mannitol (152 g/l), CKNFD trace metals (50 ml/l) and Sigma antifoam 204 (2% final). The CKNFD trace metals contained 344 mM MgCl_2_, 78.5 mM FeCl_2_, 50.5 mM MnCl_2_, 1.53 mM CuCl_2_, 1.60 mM CoCl_2_, 1.57 mM Na_2_MoO_2_, 3.23 mM ZnCl_2_, and 100 ml/liter HCl (Jakobsen et al., 2009). Bacterial growth was monitored by measuring OD_600_. Cell dry weight was calculated using a conversion factor of 0.27 g (dry weight) of cells/liter per OD_600_ unit (calculated as an average based on measurements of OD_600_ and the dry weight of cells for the fermentation trial). Due to the increase in the culture volume throughout the fermentation, the biomass, amino acids and cadaverine concentrations were corrected for the dilution as described previously (Jakobsen et al., 2009). A volume correction factor of 3.3 was used for values presented in Table 5. The actual concentrations measured in the fermentation broths were therefore accordingly lower. Samples for determination of amino acid and cadaverine titers were collected from exponential phase and throughout the cultivation (10–45 h after inoculation).

### Analyses of Growth Substrates and Products

Laminaran was quantified after acid hydrolysis to glucose (Sandbakken et al., 2018). For determination of mannitol and glucose concentrations in SWEs and culture media, samples were centrifuged and filtered through 0.2 μm syringe filters before HPLC as performed using an Aminex HPX-87-H column (BioRad Laboratories) at 45°C, and refractive index detection (RID-6A, Shimadzu). Five millimolar H_2_SO_4_ was used as mobile phase at 0.6 ml/min. Amino acids, cadaverine, and GABA samples were prepared, analyzed and quantified by the RP-HPLC method as previously described (Skjerdal et al., 1996), using pre-column derivatization with *o*-phtaldialdehyde and an increasing methanol gradient in a buffer containing 0.02 M sodium acetate, 2% tetrahydrofuran pH 5.9 at 1.0 ml/min.

## Data Availability Statement

The raw data supporting the conclusions of this manuscript will be made available by the authors, without undue reservation, to any qualified researcher.

## Author Contributions

IA planned and supervised the harvesting, extraction and analysis of seaweeds, planned initial microwell cultivations with seaweed and revised the manuscript. IN planned, performed and analyzed the fermentations with resulting products and data, and revised the manuscript. TH planned and constructed the pTH1mp-crtMN plasmid, supervised the initial analyses of terpenoid production by MGA3 (pTH1mp-crtMN), and revised the manuscript. KK performed terpenoid analysis and revised the manuscript. TB wrote the manuscript. SH planned and performed all cultivations, experimental work with hydrolyzation of seaweed extracts, performed fermentations, analyzed data and wrote the manuscript. All authors read and approved the final manuscript.

## Conflict of Interest

The authors declare that the research was conducted in the absence of any commercial or financial relationships that could be construed as a potential conflict of interest.
