# Supplementary figures and images for: Production of Value-Added Chemicals by Bacillus methanolicus Strains Cultivated on Mannitol and Extracts of Seaweed Saccharina latissima at 50°C
Source: Front Microbiol. 2020 Apr 9;11:680. doi: 10.3389/fmicb.2020.00680 (PMC7161427; doi:10.3389/fmicb.2020.00680)

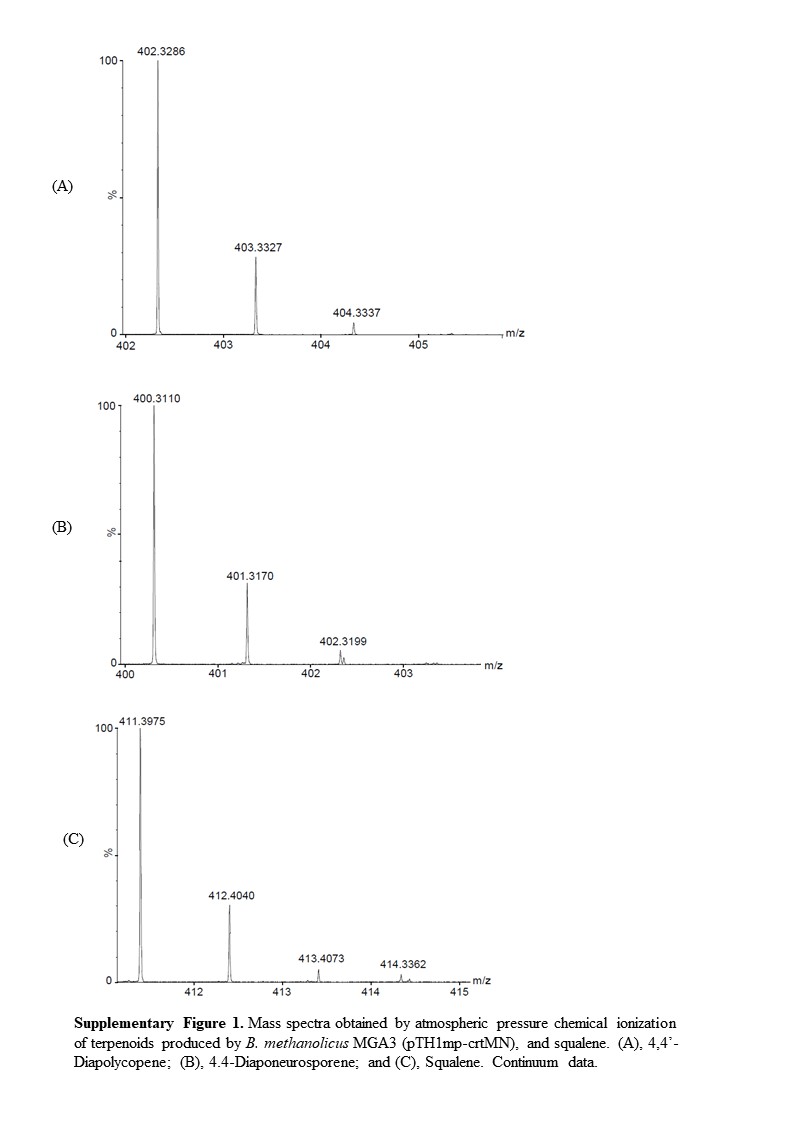

Supplement: Supplementary file 1 [file Image_1.JPEG]
